# The regulation of learning in clinical environments: A comment on ‘Beyond the self’

**DOI:** 10.1111/medu.14055

**Published:** 2020-02-13

**Authors:** Patrick Nieboer, Mike Huiskes

**Affiliations:** ^1^ Department of Orthopaedic Surgery University Medical Centre Groningen Groningen the Netherlands; ^2^ Centre for Language and Cognition University of Groningen Groningen the Netherlands

## Abstract

In this commentary, the authors highlight the importance of analyzing real time social interactions to discover strategies clinical students and residents use to self‐ and co‐regulate their learning.

Is learning in clinical workplaces deliberately regulated? If so, is it regulated by others or by the self? In this issue of *Medical Education*, Bransen et al report on a study in which they interviewed clinical students about their perceptions of learning.^1^ The authors demonstrate that students progress through three interrelated shifts during their clerkships in an intricate interplay between self‐regulated learning (SRL) and co‐regulated learning (CRL). They conclude that: ‘workplace learning, including development of SRL, always occurs in interaction with others, and that student SRL always involves engagement in CRL.’^1^ As a result, they stress that educators need to: ‘focus on facilitating and organising learners' engagement in co‐regulated learning from the start of the curriculum.’^1^

Bransen et al's results nicely outline what students want to learn (ie, their learning goals) and strategies they use to organise learning moments through participation in the clinical workplace.^1^ This builds on the tradition of Lave and Wenger, Eraut, and Billet, all of whom have given substantial attention to the issue of gaining access to relevant learning encounters in the workplace, emphasising the importance of doing so.^2^, ^3^, ^4^ Sheehan et al elaborated on their work and discovered the strategies learners use to manoeuvre in the clinical workplace effectively.^5^ According to these authors, learners need to ‘poke their nose in,’ ‘get the basics right,’ ‘offer to do things’ and undertake ‘personal reading.’^5^ Sheehan et al^5^ made the implicit strategies of the workplace explicit and accessible to students and residents to regulate their learning.

Less clear from this series of work is how students manage CRL and SRL in the day‐to‐day practice of patient care itself. More specifically, after reading Bransen et al^1^ we find ourselves wanting to know more about how students manage their expertise gaps and how they construct and recruit expertise to fill those gaps when they need to. In this commentary, we elaborate on why this is so important. We will also argue that proper understanding of CRL and SRL forces us direct our attention to the management of learning in moment‐to‐moment interactions.

Proper understanding of CRL and SRL forces us to direct our attention to moment‐to‐moment interactions

In SRL, students engage themselves in processes of testing strategies to meet their learning goals.^6^, ^7^ This is a very useful model that has been shown to be highly applicable when describing pre‐clinical learning. It is less clear, however, whether the model effectively describes what learners are able to do in more complex and unpredictable clinical learning environments.^8^ To detect learning strategies in clinical workplaces, we need to shift our focus away from studying the perceptions students hold about learning and give greater attention to the real‐time interactions that take place in the clinical workplace. It is such observations that will allow us to identify best practices and to construct instructions and learning environments that ‘facilitate and organise learners' engagement.’^1^

Clinical workplaces bring learners on to the main stage of learning: the place in which they meet patients and supervisors. This stage has important features that are distinct from those of pre‐clinical classroom‐based environments: supervisors co‐regulate learning by entrusting learners with autonomy for their patients,^9^, ^10^ and supervisors bring experience, and theoretical, procedural and practical knowledge that can directly influence learners' recruitment of SRL processes.^11^ Further, learners face the challenge of managing their learning in an environment in which patient care is the over‐riding priority.

Learning in clinical settings is embedded in collaboration, in 'joint activities'

A fine‐grained analysis of actual interactions in this environment would offer great potential as a tool to better understand SRL and CRL and to build on the insight that learning is shaped in and through interaction. Learning in clinical settings is embedded in collaboration, in ‘joint activities.’^12^ Collaboration is a coordinated effort; supervisors and learners are organised as a group, a collective team engaged in a single project that entails a *mutually* shared cognition.^13^ To experience mutually shared cognition, learners and supervisors need to attend to the same problem, know what the other does, and know what the other knows.^13^ Ideally, in clinical learning environments, learners and supervisors do not operate as separate individuals, but become collectively and jointly engaged in patient care (ie*,* together they form a cognitive unit) and in an educational alliance.^13^ Collaboration ceases to be effective at moments when members of the cognitive unit (in our case, a learner and a supervisor) fail to fulfil the requirements of mutually shared cognition.^13^ At such moments, learners and supervisors signal problems in collaboration and demonstrate repair behaviours.^12^, ^13^

Learners and supervisors do not operate as separate individuals, but become collectively and jointly engaged in patient care

To understand how learners and supervisors coordinate their joint actions during patient care, we need to look at moment‐to‐moment interactions within the context of their joint projects to determine when and how cognition about the learner's development can be furthered. The method of conversation analysis (CA) might provide a particularly useful lens through which to study such collaborative action. The fundamental tenet of CA is that concrete patterns of interaction between individuals embody information about their individual goals and offer insight into how they try to achieve those goals.^14^ We applied this approach in our work on collaboration and learning in the operating room (OR) by analysing how residents shape (self‐regulate) their learning strategies in the OR and identified four strategies used by residents to recruit expertise.^11^

Residents shape their learning strategies in the OR and use four strategies to recruit expertise

In a follow‐up study, we analysed how supervisors regulate entrustment of autonomy (ie, co‐regulate learning) and found that supervisors use nine strategies with different regulatory effects on the autonomy of the learner (Nieboer P, Huiskes M, Stevens M, Cnossen F, Bulstra SK, Jaarsma DACD. The supervisor's toolkit: strategies of supervisors to entrust and regulate autonomy of residents in the operating room. Unpublished paper, 2019). Importantly, both residents and supervisors demonstrated variation in the use and frequency of strategies within and between clinical procedures,^11^ (Nieboer P, Huiskes M, Stevens M, Cnossen F, Bulstra SK, Jaarsma DACD. The supervisor's toolkit: strategies of supervisors to entrust and regulate autonomy of residents in the operating room. Unpublished paper, 2019) which suggests a need to further understand the tools learners and supervisors engage during moment‐to moment interactions as procedures unfold.

Supervisors use nine strategies with different regulatory effects on the autonomy of the learner

Juxtaposing these findings with those of Bransen et al^1^ indicates a path through which we can identify best practices for both supporting SRL and improving supervisors' capacity to co‐regulate learning. Further exploration of how role‐model supervisors and role‐model residents apply both SRL and CRL tools to optimise learning during patient care will help to make workplace‐based learning processes explicit, thereby providing guidance on how we should think about how we can collectively begin to organise ‘learners' engagement in co‐regulated learning.’^1^
